# Surgical tray optimization: a prospective and survey-based evaluation of environmental and economic outcomes

**DOI:** 10.1007/s00464-025-12499-2

**Published:** 2026-01-23

**Authors:** M. M. M. Eussen, R. M. Brandwijk, R. C. Reising, M. L. Kimman, P. Nakhate, Y. van der Meer, T. Stobernack, N. D. Bouvy

**Affiliations:** 1 Department of Surgery, Maastricht University Medical Center, Maastricht, The Netherlands; 2 NUTRIM School of Nutrition and Translational Research in Metabolism, Maastricht University, Maastricht, The Netherlands; 3 Aachen-Maastricht Institute for Bio-based Materials (AMIBM), Maastricht University, Maastricht, The Netherlands; 4 Department of Clinical Epidemiology and Medical Technology Assessment (KEMTA), Maastricht University Medical Centre, Maastricht, The Netherlands; 5 Department of Intensive Care Medicine, Radboud University Medical Centre, Nijmegen, The Netherlands; 6 Department of Surgery, Leiden University Medical Center, Albinusdreef 2, 2333 ZG Leiden, The Netherlands

**Keywords:** Surgical tray optimization, Reusable instruments, Life cycle assessment, Cost analysis, Sustainable surgery

## Abstract

**Background:**

Operating rooms are resource-intensive environments. While reusable instruments are more sustainable than disposables, sterilization and reprocessing of unused instruments in the trays generate unnecessary environmental and financial burdens. Surgical tray optimization—removal of rarely used instruments—may reduce these impacts. Current evidence is fragmented, with no standard method to identify unused instruments and limited integration of environmental and cost analyses.

**Methods:**

We prospectively recorded instrument use for the Major General Surgery tray (94 reusable instruments) in one academic hospital in the Netherlands during 162 surgical procedures across multiple disciplines (November 2023–May 2024). Instruments used in < 20% of cases were considered for removal by (1) prospective clinical observation and (2) retrospective staff survey (surgeons, residents, scrub nurses). Life cycle assessment (LCA) and activity-based costing quantified the carbon footprint and costs.

**Results:**

On average, 19 instruments were used per case; 10 were never used. Observation-based optimization reduced tray size and weight by 54%, lowering costs by 55%. Survey-based review achieved smaller reductions (22% in number, 18% in weight, 21% in cost), with surgeons supporting more removals than scrub nurses. The original tray generated 1.25 kg CO_2_-eq per use. Optimization reduced emissions by 0.91 and 2.69%, corresponding to thresholds of < 10 and < 20% use, respectively.

**Conclusion:**

In multi-specialty settings, tray optimization offers substantial cost savings and modest but cumulative environmental benefits. Clinical observation identified greater reductions than staff reviews. Combining LCA with cost analysis can provide a transparent framework to guide sustainable surgical practice.

**Supplementary Information:**

The online version contains supplementary material available at 10.1007/s00464-025-12499-2.

Modern healthcare faces two urgent challenges: reducing its carbon footprint and managing rising costs [1–4]. The climate crisis is the greatest health threat of our time, yet paradoxically healthcare itself contributes about 4% of global greenhouse gas (GHG) emissions and up to 6–10% in some countries [3, 5–7]. Meanwhile, healthcare spending rose from 8.8 to 9.7% of GDP between 2019 and 2021 [8, 9]. These dual pressures demand reforms that align clinical care with sustainability and cost-effectiveness, ensuring that healthcare protects health without undermining planetary and economic stability.

The operating room (OR) is a key target for reformation, as it disproportionately consumes hospital resources and contributes to environmental pollution and healthcare costs [10–14]. Surgical care often follows a linear “take-make-dispose” model—in which raw materials are taken from the environment, manufactured into single-use instruments, and discarded after one procedure—increasing both waste and expense [15]. In contrast, a circular economy extends material lifecycles by improving resource efficiency and reducing environmental harm, guided by the10R framework: refuse, reduce, rethink, reuse, repair, refurbish, remanufacture, repurpose, recycle, and recover [16]. For instance, replacing disposables with reusable alternatives lowers emissions and costs, since single-use items require resource-intensive production and disposal, whereas reusable instruments can be reprocessed [15]. Circular strategies in the OR offer a practical path to more sustainable, cost-effective surgical care.

However, sterilization and cleaning can account for up to 90% of the carbon footprint of reusable instruments [17–19]. Surgical tray optimization—reducing the number of instruments in standardized sets—can mitigate this, since entire trays must be processed after each procedure regardless of actual use. Trays often grow over time due to surgeon preferences, leaving many tools unused. A recent review found that over half the instruments could be removed, reducing carbon footprint by up to 66% and cutting costs by 32–78% [20]. Tray optimization might therefore be a high-impact strategy to enhance the sustainability of reusable surgical equipment.

Despite this potential, optimization lacks standardization, and the best method to identify unused instruments remains uncertain [20]. Some studies support direct observation during procedures; others favor clinician-led tray reviews [20]. However, no studies have not directly compared the environmental and financial outcomes of different optimization strategies. Moreover, many rely on hypothetical models not aligned with real workflows, such as sterilization machine capacity. Understanding how different approaches influence both environmental and financial results is essential to maximize the impact.

This study is the first to quantify unused instruments in multi-use surgical trays and assess the environmental and financial impact of removing them through two approaches: clinical observation and retrospective clinician review. Unlike previous research limited to single procedures, it analyzes a tray used across multiple specialties and involves key stakeholders to ensure results reflect clinical practice. By comparing these methods, this study aims to support evidence-based strategies for sustainable surgical tray design.

## Methods

This study was conducted at Maastricht University Medical Centre (MUMC+) in the Netherlands. The study was approved by the Institutional Review Board of the MUMC+ as non-medical research in accordance with the Dutch Human Subjects Act (Non-WMO) (METC 2025-0042). The study adhered to the STROBE guidelines for observational research [21].

**Fig. 1:**
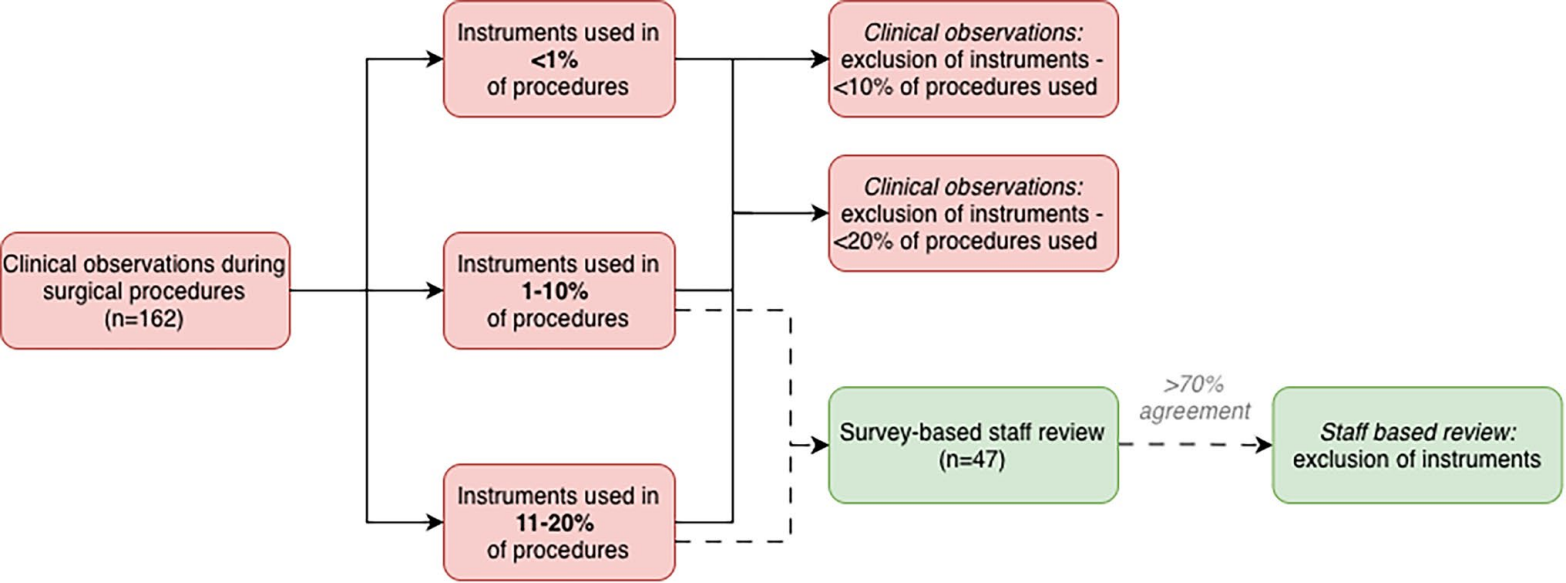
Process of instrument selection and exclusion based on clinical observation and staff review

Following prior research, instruments used in fewer than 20% of procedures (1–10% and 11–20%) were identified as candidates for removal [20]. We applied these thresholds to the Major General Surgery (MGS) tray, a 94-instrument reusable set used across multiple disciplines (Appendix A). To identify instruments for removal, we used two approaches: (1) clinical observation during surgical procedures and (2) survey-based staff review (surgeons and scrub nurses). Figure 1 outlines the evaluation process. We quantified the environmental and economic impact of the original tray compared with optimized tray compositions.

### Data collection: identifying instruments for removal

#### Clinical observation of instrument use during surgical procedures

From November 2023 to May 2024, instrument use was prospectively recorded during randomly selected surgeries involving the MGS tray. We created a checklist of all instruments and their quantities to ensure accurate tracking.

After each procedure, the scrub nurse separated the instruments that had been used and informed the research team. The research team comprised one researcher and medical students from the Department of General Surgery. Instruments directly handled by the surgeon or resident were also classified as “used.” The research team documented the number of instruments used, the surgery date and time, and the operating room number. We later reviewed surgery reports to determine the procedure type and identify the attending surgeon(s), resident(s), and scrub nurse(s).

We entered data into Microsoft Excel (Microsoft, 2024) and analyzed them in SPSS© version 29.0. Descriptive statistics included means and standard deviations for continuous variables and frequencies for categorical variables. The required sample size (*n* = 137) was determined a priori with VassarStats, assuming a power of 0.90 and *α* = 0.05.

#### Survey-based staff review of instrument removal

In the second phase, we assessed clinician input on instrument removal through an online survey. Based on our observational data, instruments used in 1–10% or 11–20% of procedures were included for review, while those used in fewer than 1% of cases were automatically designated for removal, as they were considered clinically irrelevant (Fig. 1). This rule reduced survey burden and ensured consistency with the observation-based method.

The survey was created in Google Forms, distributed to all surgical specialties using the tray, and included images of each instrument. A reminder followed after two weeks, and responses were collected for another two weeks. Surgeons and scrub nurses indicated whether they supported removal, and for duplicate instruments, how many could be eliminated. Instruments with > 70% agreement for removal were excluded. The full survey is provided in Appendix B.

### Environmental and financial impact assessment

As this study aims to reflect real clinical practice, we based our assumptions on expert opinion. Instruments were modeled to be reused at least 500 times—a conservative estimate, as many have already been in use for over ten years. Experts also advised which processes would be affected by reducing tray size (Appendix C) and noted that instruments are processed in two baskets for cleaning and sterilization (hinged and non-hinged). Both baskets remained necessary even when the tray size was reduced by half.

#### Weight of the tray

We used a weighing scale to record the weight in grams of the entire tray (including net) and of each individual instrument. For identical instruments, we calculated the mean weight from multiple measurements, as variations were observed between instruments.

#### Life cycle assessment (LCA)

**Fig. 2:**
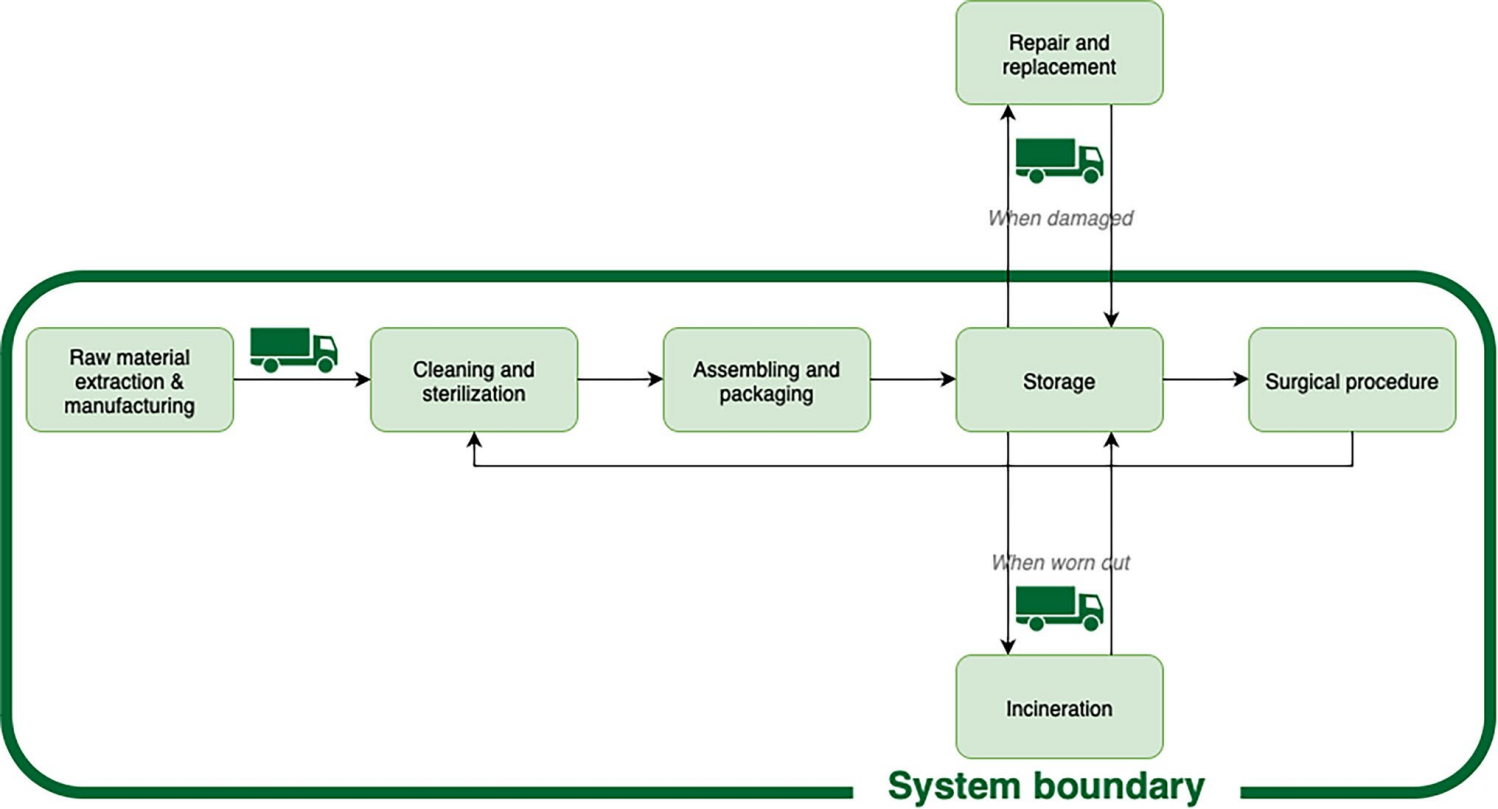
System boundaries in the life cycle assessment of the original and optimized major general surgery tray

We conducted a life cycle assessment (LCA) in accordance with ISO 14040/14044 guidelines [22, 23]. The functional unit was one use of the original reusable MGS tray and its reduced versions, in a single surgical procedure. The scope was cradle-to-grave, encompassing the production, transport, storage, use, sterilization, packaging, and disposal phases of the tray (Appendix D). Figure 2 illustrates the system boundaries.

To build the production inventory, we contacted manufacturers for information on origin and transportation routes. Sterilization inventory came from structured interviews with the hospital’s Central Sterilization Department and technical specifications of sterilization equipment. Waste processing information was derived from the literature [24]. A comprehensive overview of the inventory and assumptions made during its construction is provided in Appendix D and E [24–30].

We processed inventory data in ActivityBrowser (version 2.11.2), using the Ecoinvent database (version 3.9.1) for all processes, and assessed environmental impacts with the World ReCiPe 2016 Midpoint (Hierarchist) method (version 1.1) [31–33].

#### Activity-based cost analysis

We conducted an activity-based cost analysis to evaluate the original tray and its reduced versions for a single surgical procedure. Analysis mapped the full instrument pathway across lifecycle stages identified in the LCA, developed in close collaboration with surgeons, scrub nurses, and central sterilization staff.

**Fig. 3:**
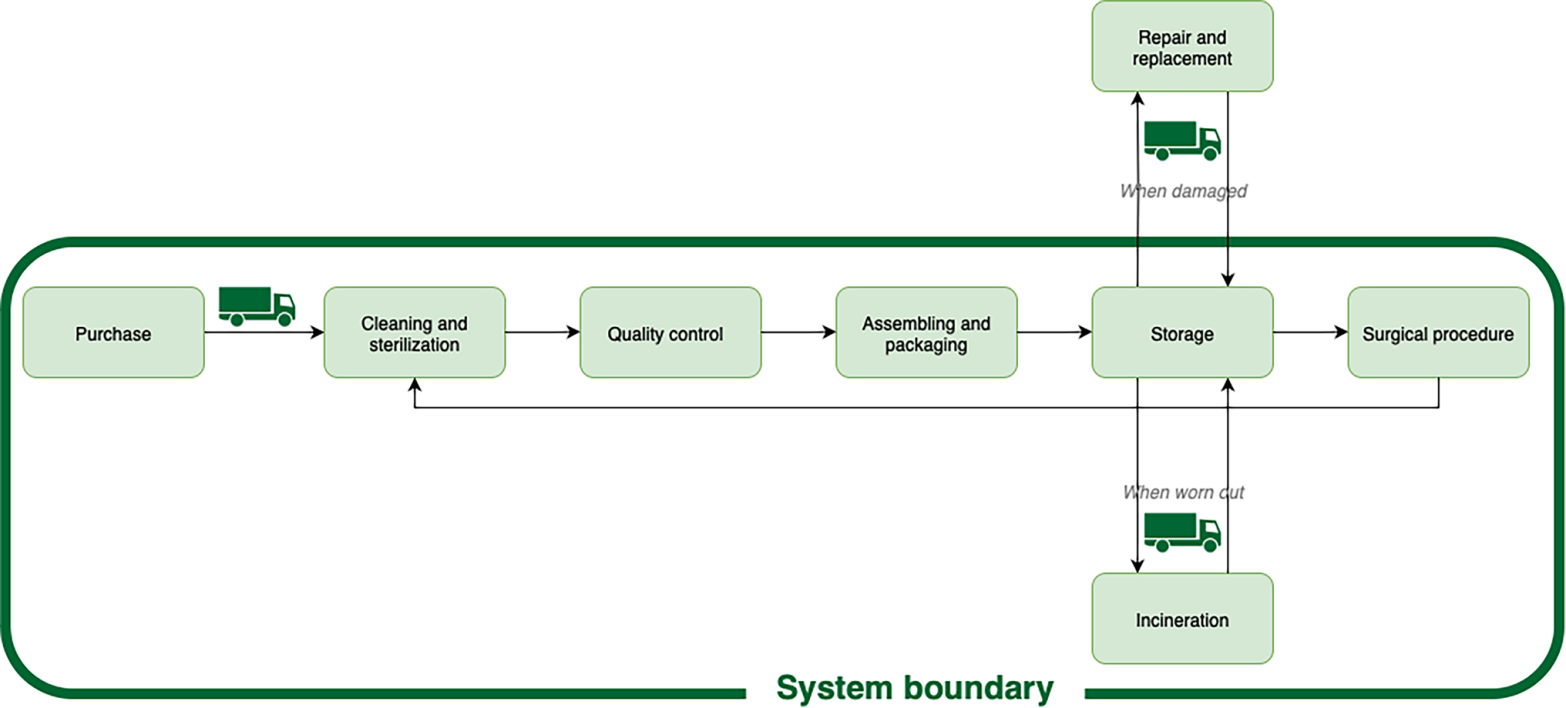
Process flow and system boundaries in the activity-based cost analysis

The analysis included the following components: (1) instrument purchase price; (2) cleaning and sterilization; (3) quality control and maintenance; (4) assembly and packaging; (5) storage; and (6) disposal of instruments no longer suitable for reuse. Instrument repair and replacement were excluded, as these are handled by the manufacturer and covered in the purchase price. Figure 3 illustrates the processes that were included and excluded in the analysis.

The analysis focused on short-term, per-tray costs without discounting, as all activities occurred within a one-year time horizon. All costs are reported in 2025 euros. We adjusted the costs collected in 2024 to July 2025 values using the consumer price index of Statistics Netherlands (CBS). We applied a hierarchical data collection strategy. We obtained primary data from institutional records, including procurement databases, sterilization department logs, and manufacturer data, supplemented by input from procurement officers and personnel from the sterile services and finance departments. When institutional data were unavailable, we relied on published literature or, where necessary, expert opinion. Full details are provided in Appendix C.

## Results

### Clinical observation of instrument use during surgical procedures

#### Procedure and user characteristics

Between November 2023 and May 2024, 162 surgical procedures were included for observation. The MGS tray was used in 205 procedures during this period; however, 43 cases were excluded due to lack of research team notification or premature tray removal.

Included procedures encompassed breast, minor, endocrine, robotic, and hernia surgeries (Appendix F). The most performed procedures were unilateral lumpectomy or mastectomy with sentinel node biopsy (*n* = 48, 29%) or without (*n* = 20, 12%). Most surgeries were performed by general surgery staff or residents, with some involving plastic surgery (*n* = 26) or gynecological surgery (*n* = 1). A total of 36 surgeons and 20 residents used the tray, with usage per individual ranging from 1 to 38 procedures. All resident-involved cases were supervised by a staff surgeon. A single scrub nurse participated in 156 cases, while two scrub nurses were involved in six. Overall, 46 scrub nurses used the tray, with individual usage ranging from 1 to 13 times.

#### Instrument usage

An average of 19 (± 7) instruments were used per case. Detailed results are presented in Table 1 and Appendix A.

**Table 1:** Summary of outcomes for the original and optimized major general surgery instrument tray, using two optimization approaches: clinical observation and retrospective survey-based staff review

	Original major general surgery tray	Clinical observation during surgical procedures				Survey-based staff review					
		< 10% of cases used		< 20% of cases used		Total		Based on surgeons		Based on scrub nurses	
			Reduction (%)		Reduction (%)		Reduction (%)		Reduction (%)		Reduction (%)
Number of instruments	94	51	45.74	43	54.26	73	22.34	64	31.91	71	24.47
Weight (grams)	4309	2443	43.31	1969	54.31	3516	18.40	2977	30.91	3383	21.49
Carbon footprint (kg CO_2_eq)	1.2514	1.2246	2.14	1.2178	2.69	1.2400	0.91	1.2323	1.53	1.2381	1.06
Costs (euros)	43.80	24.21	44.73	20.11	54.09	34.67	20.85	30.42	30.54	33.78	22.87

### Survey-based staff review of instrument removal

#### Respondent characteristics

A total of 47 staff members completed the survey, including 20 attending surgeons or residents and 27 scrub nurses. The majority of respondents were women (*n* = 34; 72.3%) and had worked at the hospital for more than 10 years (*n* = 22; 46.8%). Most reported using the MGS tray either weekly (*n* = 16; 34.0%) or multiple times per week (*n* = 14; 29.8%). Details are provided in Appendix G.

### Outcomes for tray optimization

#### Number of instruments

Procedural observations showed that removing instruments used in fewer than 10% of surgeries would reduce the tray size by 45%. Applying a 20% threshold would result in a 54% reduction. Based on staff survey responses—after excluding instruments used in fewer than 1% of cases—the optimized tray would include 73 instruments, representing a 22% reduction. When considering only surgeons’ and residents’ responses, the tray could be reduced by 32%, whereas optimization based on scrub nurse responses alone would result in a 26% reduction. Detailed results are presented in Table 1 and Appendix A.

#### Weight of the tray

The full instrument set weighed 4309 g; including the tray, 5257 g in total. Removing instruments used in < 10% of cases, based on clinical observations, reduced the weight by 43%; applying a < 20% threshold reduced it by 54%. The survey-based optimization resulted in a tray weight of 3516 g (18% reduction). Full results are provided in Table 1.

#### Carbon footprint

**Fig. 4:**
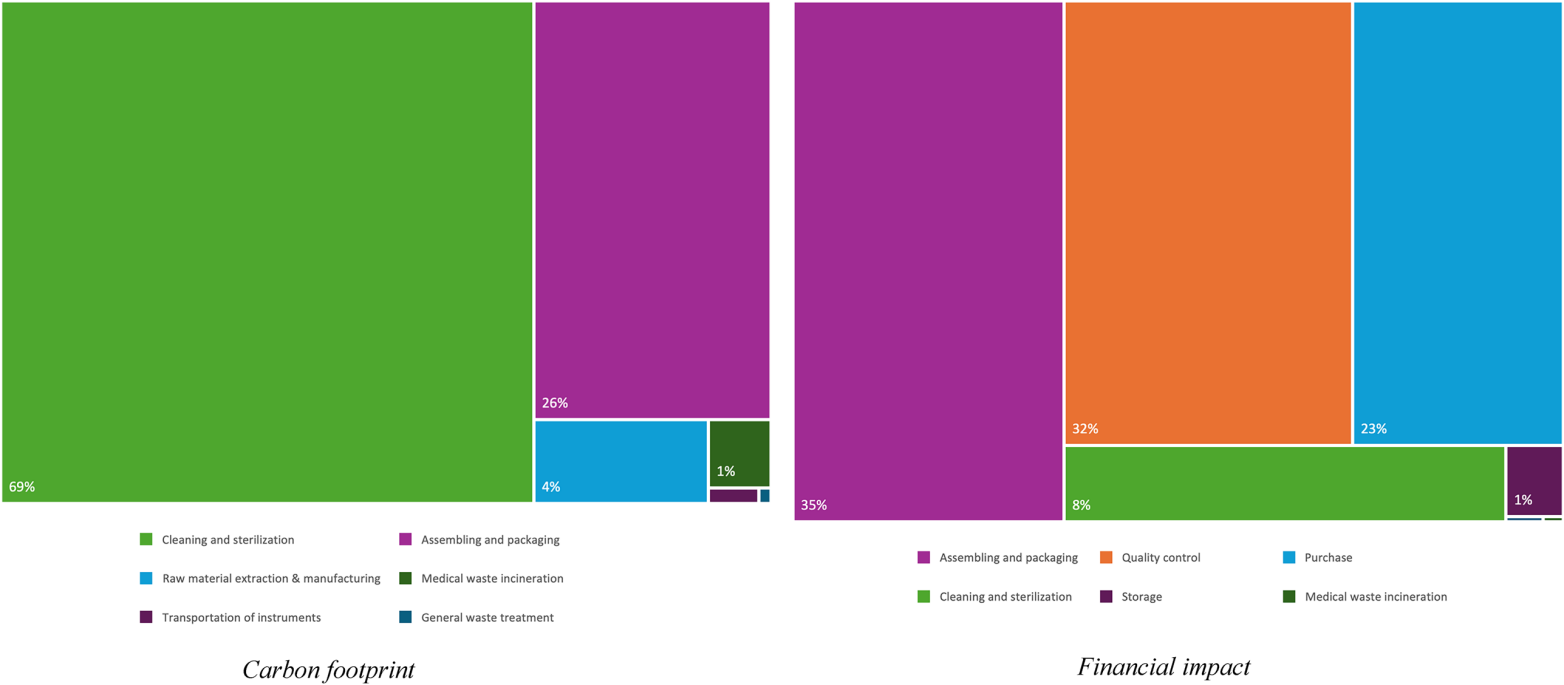
Contribution of processes to the carbon footprint (left) and the costs (right) of the original Major General Surgery instrument tray

The lifecycle of one original MGS tray was 1.25 kg carbon dioxide equivalent (CO_2_-eq) per surgery. Main contributors were cleaning and sterilization (69%), followed by assembling and packaging with blue wraps (26%). Details are presented in Fig. 4 and Appendix C.

Optimizing the tray reduced CO_2_-eq by 0.91–2.69%. The largest decreases were achieved by removing instruments used in fewer than 10 and 20% of cases, based on clinical observations, resulting in reductions of 2.14 and 2.69%, respectively. Full results are presented in Table 1 and Appendix C.

#### Financial impact

Assuming 500 reuses, the original MGS tray cost was €43.80 per surgery. Main contributors were assembly and packaging (35%), quality control (32%), and purchase (23%). Assembly and packaging costs were driven predominantly by labor (96%), alongside the use of blue wraps, while quality control costs were entirely labor-based. Because these activities scale with the number of instruments handled, reducing tray contents directly decreases processing time and therefore lower labor costs. Details are presented in Fig. 4 and Appendix C.

The largest cost reduction (54%) was achieved by removing instruments used in fewer than 20% of cases, based on clinical observations. Survey-based staff reviews yielded a 21% decrease, while surgeon-only and scrub nurse-only reviews reduced costs to €30.42 (30.54%) and €33.78 (22.87%) per surgery, respectively. Full results are provided in Table 1.

Overall, cost savings were driven primarily by reductions in labor time for assembly, packaging, and quality control—tasks that scale directly with the number of instruments handled. In contrast, sterilization costs remained largely unchanged: although cleaning labor may decrease slightly due to fewer instruments being brushed and inspected, energy, water, and detergent use remained constant because the sterilizer load did not change. Additional information on which cost components change—and how these changes were calculated—is provided in Appendix C.

## Discussion

Operating rooms are resource-intensive environments, contributing disproportionately to healthcare’s environmental and financial burden [1–4]. Reusable surgical instruments are generally more sustainable than disposables, yet their sterilization and maintenance carries substantial environmental and economic impacts, particularly when instruments remain unused [17–19]. Tray optimization offers a potential solution, but evidence is fragmented: no universal method exists to identify unused instruments, most studies address single-specialty trays, and few assess environmental and financial outcomes together [20]. This study addresses these gaps by (1) quantifying unused instruments in a multi-specialty surgical tray, and (2) directly comparing ecological and financial outcomes of two optimization strategies: prospective clinical observation and retrospective staff review.

On average one-fifth of the instruments in the tray were used during observed procedures, with 10 never used. Applying a < 20% usage threshold via clinical observation reduced instrument numbers and tray weight by more than half, cutting per-procedure costs by 55%. Survey-based staff review produced smaller reductions (22% in number, 21% in cost), with surgeons supporting more removals than scrub nurses. The original tray generated 1.25 kg CO_2_-eq per use; optimization lowered this by 0.91–2.69%, with the largest decreases achieved when removing instruments used in fewer than 10% (2.14%) or 20% (2.69%) of cases. Overall, clinical observation proved more effective, identifying more unused instruments and yielding greater economic and ecological improvements than surveys.

These findings align with our previous systematic review showing that over half of instruments in surgical trays can often be removed [20]. The present study confirms the pattern in a multi-specialty tray, with reductions > 50% in some scenarios, extending evidence beyond single-specialty contexts. Given higher case volumes, optimizing multi-use trays may therefore yield substantial cumulative benefits across repeated sterilization cycles, even if single-tray environmental gains remain modest.

The greater reductions from clinical observation support earlier claims that prospective methods capture actual usage more accurately than surveys, which are prone to recall bias—often influenced by perceived necessity or professional risk attitudes—as well as cognitive and implicit biases [34, 35]. In our study, scrub nurses retained more instruments, possibly reflecting concerns about preparedness, while surgeons readily supported removing rarely used tools. To our knowledge, no prior studies have compared these perspectives, highlighting an area for future research. Interestingly, several surgeons noted postoperatively that even fewer instruments might suffice, though this was not reflected in their survey responses. Achieving lasting change therefore requires both accurate identification and acceptance among daily users, underscoring the need to involve all stakeholders.

Previous LCAs showed that decontamination and sterilization dominate the carbon footprint of reusable instruments [17–19]. Our findings align: cleaning and sterilization accounted for the largest share of the tray’s footprint, followed by blue-wrap packaging. Although reducing instrument numbers can lower per-procedure emissions and costs, proportional savings are constrained by operational factors, including the inability to adjust tray size for more efficient machine loading. In our setting, all instruments must be transferred from the initial tray into two fixed-size decontamination baskets—one hinged and one non-hinged—which are placed in the sterilizer. Both baskets remained necessary even when the number of instruments was halved, and therefore the reduced instrument volume did not create additional space in the sterilizer for other trays. Consequently, the loading efficiency of the machine did not change, and no reductions in water, energy, or detergent use per processed surgical tray were observed. As a result, only minimal carbon footprint reduction was achieved. Designing smaller baskets that allow more efficient machine loading could reduce water, detergent, energy, and equipment use per cycle, yielding additional environmental and financial savings. This underscores the importance of involving multiple stakeholders when modeling outcomes to reflect real-world logistics. While single-tray reductions were modest, scaling across multiple trays and thousands of cycles could result in meaningful cumulative benefits.

Our results identify major contributors to the carbon footprint of surgical trays and provide insight in complementary strategies beyond instrument reduction. Blue wraps accounted for a quarter of emissions and could be replaced with reusable rigid containers [36, 37]. Although waste processing contributes only a small share, streamlining waste streams may help; in our study, metal instruments were discarded as high-impact medical waste, whereas dedicated recycling could yield additional reductions in line with the 10R framework [16, 38, 39]. While tray optimization achieved only moderate emission reductions, the identification of major contributors with available sustainable alternatives offers clear opportunities to further lower environmental impact.

Economically, our results fall within reported savings (32–78%) [20]. Instrument purchase price was a major cost driver, suggesting that extending instrument lifespan beyond the 500 reuses assumed here could yield greater savings. Our costing approach aligned with LCA boundaries, providing a transparent framework for assessing environmental and economic outcomes together. This dual perspective is crucial, as sustainability measures that also cut costs strengthen the case for adoption in healthcare, where financial factors remain key [40].

An underexplored aspect of tray optimization is the fate of permanently removed instruments—whether retained for rare cases, reintroduced as disposables, or redistributed to other sets. As sterilization drives much of the footprint, these choices have direct sustainability implications and should be investigated further. Partial tray reductions also pose logistical challenges: full sets may still be opened for a single missing item, limiting gains unless addressed with modular packaging or individually wrapped instruments. Above all, patient safety remains paramount, and future research should evaluate intraoperative safety alongside environmental and economic outcomes.

This study has several limitations. First, being conducted in a single academic hospital may limit generalizability, as tray composition, surgical practices, and sterilization infrastructure differ across institutions. Second, we did not assess clinical outcomes such as intraoperative delays or patient safety after instrument removal. Third, survey participation was voluntary, and non-respondents may hold different views. Both the LCA and costing analyses relied on primary data, literature, and expert opinion; assumptions such as instrument lifespan may affect absolute estimates. Finally, our environmental results reflect the Dutch context and may not apply directly to settings with different energy sources, waste systems, or procurement chains.

## Conclusion

This study demonstrates that surgical tray optimization in a multi-specialty context can substantially reduce financial burdens and deliver modest but consistent environmental gains, with the most pronounced benefits achieved through prospective clinical observation of instrument use. While survey-based staff reviews also produced savings, they identified fewer removable instruments and delivered smaller environmental gains, with surgeons agreeing to remove more instruments than scrub nurses.

By combining LCA with activity-based costing, we provide evidence that reductions in tray size translate into both lower costs and measurable decreases in carbon footprint. Although small at the tray level, these reductions accumulate across thousands of sterilization cycles, supporting tray optimization as a practical, evidence-based strategy for sustainable surgical practice. Integrating real-world usage data with clinician engagement and sterilization logistics offers a clear, actionable pathway to greener, leaner, and more cost-efficient surgery.

## Supplementary Information

Below is the link to the electronic supplementary material.

Supplementary file1 (PDF 175 KB)—Appendix A: Instrument list and utilization rates for the major general surgery tray

Supplementary file2 (PDF 111 KB)—Appendix B: Survey sent to surgeons and scrub nurses on instrument removal

Supplementary file3 (XLSX 40 KB)—Appendix C: Overview of data sources and calculations for life cycle assessment and activity-based cost analysis

Supplementary file4 (PDF 122 KB)—Appendix D: Inventory of the life cycle assessment for using the current instrument set to conduct one surgery

Supplementary file5 (PDF 74 KB)—Appendix E: Overview of assumptions for life cycle assessment

Supplementary file6 (PDF 76 KB)—Appendix F: Types and frequencies of observed procedures

Supplementary file7 (PDF 84 KB)—Appendix G: Characteristics of staff surveyed for instrument review
