# Supplementary material for: Surgical tray optimization: a prospective and survey-based evaluation of environmental and economic outcomes
Source: Surg Endosc. 2026 Jan 23;40(4):3080–9. doi: 10.1007/s00464-025-12499-2 (PMC13053359; doi:10.1007/s00464-025-12499-2)
Supplement: Supplementary file 1 — Supplementary file1 (PDF 175 KB)—Appendix A: Instrument list and utilization rates for the major general surgery tray [file 464_2025_12499_MOESM1_ESM.pdf]

Appendix A: Instrument list and utilization rates for the major general surgery tray

Instrument	Size (mm)	Quantity in tray	Number of cases where instrument was used (%)	Survey-based staff review								
				Total			*Based on surgeons*			*Based on scrub nurses*		
				Agreement on removal	Number of instruments removed from tray	Number of instruments in optimized tray	Agreement on removal	Number of instruments removed from tray	Number of instruments in optimized tray	Agreement on removal	Number of instruments removed from tray	Number of instruments in optimized tray
Needle holders												
Baby crilewood	150	1	72 (44%)			1			1			1
Crilewood	180	1	53 (33%)			1			1			1
Mathieu	170	1	161 (99%)			0			0			0
Mayo-hegar, straight	180	1	73 (45%)			1			1			1
Retractor												
Abdominal retractor	210	1	162 (100%)			0			0			0
Adson blunt	200	1	142 (88%)	15 (32%)		1	8 (40%)		1	7 (26%)		1
Crile nerve hook	200	1	161 (99%)			0			0			0
Doyen	35x60	1	160 (99%)	29 (62%)		0	15 (75%)	1	0	14 (52%)		1
Kocher-Langenbeck	215, 41x11	2	113 (70%)			2			2			2
Langenbeck	60x20	2	129 (80%)	15 (32%)		2	6 (30%)		2	9 (33%)		2
Langenbeck	210, 30x14	2	112 (69%)			2			2			2
Liver	700x300	2	159 (98%)	32 (68%)		2	14 (70%)	2	0	18 (67%)		2
Middeldorf	215, 20x22	2	112 (69%)			2			2			2
Middeldorf	235, 28x28	2	107 (66%)			2			2			2
Volkmann 4-prong sharp	220	2	69 (43%)			2			2			2

Wound retractor 4-prong sharp	170	2	91 (56%)			2			2			2
Scissors												
Mayo dissecting, curved	-	1	18 (11%)			1						1
Metzenbaum	145	1	131 (81%)	25 (53%)		1	8 (40%)			17 (63%)		1
Metzenbaum	180	1	47 (29%)			1						1
Scissors straight, blunt	145	1	142 (88%)	31 (66%)		1	14 (70%)	1	0	17 (63%)		1
Forceps												
Adson, surgical with plateau	120	2	140 (86%)	25 (53%)		2	10 (50%)		2	15 (56%)		2
Allis tissue forceps, atraumatic	200	1	141 (87%)	7 (15%)		1	2 (10%)		1	5 (19%)		1
Anatomical tissue	145	2	121 (75%)			2			2			2
Babcock	215	1	158 (98%)	17 (36%)		1	9 (45%)		1	8 (30%)		1
Baby crile (Christoph)	-	6	135 (83%)	41 (87%)	2	4	16 (80%)	2	4	25 (93%)	2	4
Backhaus towel clamp	135	1	160 (99%)			0			0			0
Craford	240	2	158 (98%)	18 (38%)		2	7 (35%)		2	11 (41%)		2
Crile	160	2	153 (94%)	32 (68%)		2	17 (85%)	2	0	15 (56%)		2
Dandy	-	1	161 (99%)			0			0			0
Debakey	150x2	2	118 (73%)			2			2			2
Debakey	200x2	2	110 (68%)			2			2			2
Debakey	200x3.3	2	107 (66%)			2			2			2
DeBakey dissecting	180	1	140 (86%)	3 (6%)		1	3 (15%)		1	0 (0%)		1
Dixon-Lovelace	160	4	146 (90%)	31 (66%)		4	17 (85%)	4	0	14 (52%)		4
Foerster-Ballenger, curved	240	1	148 (91%)	31 (66%)		1	11 (55%)		1	20 (74%)	1	0
Foerster-Ballenger, straight	180	1	55 (34%)			1			1			1
Gillies	155	2	41 (25%)			2			2			2
Halsted mosquito	125	6	89 (55%)[1]	36 (77%)	2	4	11 (55%)		6	25 (93%)	2	4
Kocher	240	2	154 (95%)	36 (77%)	2	0	14 (70%)	2	0	22 (81%)	2	0
Ligature clip applier, double curved	150	1	149 (92%)	21 (45%)		1	17 (85%)	1	0	11 (41%)		1
Nissen artery curved	185	4	141 (87%)	11 (23%)		4	9 (45%)		4	23 (85%)	1	3
Pean hemostatic	140	2	76 (47%)			2			2			2
Rochester pean, straight	200	1	159 (98%)	38 (81%)	1	0	17 (85%)	1	0	21 (78%)	1	0
VGS link	-	1	158 (98%)	21 (45%)		1	10 (50%)		1	11 (41%)		1
Rest												
Baby poole suction tube	5	1	150 (93%)	29 (62%)		1	9 (45%)		1	20 (74%)	1	0
Bowl polypropylene 250cc	-	3	84 (52%)[2]	42 (89%)	1	2	20 (100%)	1	2	22 (81%)	1	2
Bowl polypropylene 60cc	-	2	137 (85%)	42 (89%)	2	0	18 (90%)	2	0	24 (89%)	2	0
Diathermy cable ERBE		1	122 (75%)			1			1			1

Drainage needle for exudrain	-	1	161 (99%)			0			0			0
Hemolock clip applier small, medium	200	1	156 (96%)	22 (47%)		1	11 (55%)		1	11 (41%)		1
Hemolock clip applier, small	200	1	120 (74%)			1			1			1
Redon needle Ch8	-	1	161 (99%)			0			0			0
Redon needle Ch12	-	1	161 (99%)			0			0			0
Scalpel handle No. 3	-	1	25 (15%)			1			1			1
Scalpel handle No. 4	-	1	148 (91%)	22 (47%)		1	15 (75%)	1	0	7 (26%)	1	1
Speculum	175	2	161 (99%)	39 (83%)	2	0	16 (80%)	2	0	23 (85)	2	0
**Total**		**94**				**73**			**64**		**70**	

	Instruments never used
	Instruments used 1-10% of the cases
	Instruments used 11-20% of the cases
	Consensus >70%

[1] Based on 6 instruments, in majority of the cases only 4 instruments were used.
[2] Based on 3 instruments, in majority of the cases only 2 instruments were used.
