# Supplementary material for: Surgical tray optimization: a prospective and survey-based evaluation of environmental and economic outcomes
Source: Surg Endosc. 2026 Jan 23;40(4):3080–9. doi: 10.1007/s00464-025-12499-2 (PMC13053359; doi:10.1007/s00464-025-12499-2)
Supplement: Supplementary file 2 — Supplementary file2 (PDF 111 KB)—Appendix B: Survey sent to surgeons and scrub nurses on instrument removal [file 464_2025_12499_MOESM2_ESM.pdf]

Appendix B: Survey sent to surgeons and scrub nurses on instrument removal

This survey was originally distributed in Dutch and has been translated into English for this publication.

Section 1 – General questions

1 How would you describe your gender?

- o Male
- o Female
- o Prefer not to say
- o Prefer to describe myself as…

2 What is your current position?

- o Surgical resident
- o Fellow
- o Attending (0-5 years of experience)
- o Attending (6-10 years of experience)
- o Attending (>10 years of experience)
- o Scrub nurse in training
- o Scrub nurse (0-5 years of experience)
- o Scrub nurse (6-10 years of experience)
- o Scrub nurse (>10 years of experience)

Section 2 – Background information (medical doctors)

This section was displayed only to participants who selected "Surgical resident," "Fellow," or "Attending" in Q2.

3 What is your medical speciality?

- o General surgery
- o Gynaecology
- o Plastic surgery
- o Urology
- o Other… (please specify)

4 How long have you been employed at MUMC+?

- o 0-5 years
- o 6-10 years
- o >10 years

5 How often do you use the Major General Surgery tray?

- o Daily
- o Multiple times a week
- o Weekly
- o Multiple times a month

- Less than once a month

Section 3 – Background information (scrub nurses)

This section was displayed only to participants who selected "Scrub nurse in training" or "Scrub nurse" in Q2.

6 To which cluster are you assigned to?
- Yellow
- Blue
- Green
- Red

7 How long have you been employed at MUMC+?
- 0-5 years
- 6-10 years
- >10 years

8 How often do you use the Major General Surgery tray?
- Daily
- Multiple times a week
- Weekly
- Multiple times a month

Section 4 – Instruments used in less than 10% of cases

For each instrument listed below, please indicate whether you agree with its removal from the tray:

9 Doyen retractor (35x60 mm)
10 Liver Retractor (700x300mm) (2 pieces)
11 Babcock forceps (215mm)
12 Crafoord forceps (240mm) (2 pieces)
13 Crile forceps (160 mm) (2 pieces)
14 Dixon-Lovelace forceps (160mm) (4 pieces)
15 Foerster-Ballenger forceps, curved (240 mm)
16 Kocher forceps (240mm) (2 pieces)
17 Ligature clip applier, double curved (150 mm)
18 Rochester pean, straight (200mm)
19 VGS forceps, link
20 Spuculum (175mm) (2 pieces)
21 Hemolock clip applier small, medium
22 Scalpel handle No. 4
23 Baby poole suction tube (5mm)

Answer options for each instrument listed above:
- Yes, remove this instrument from the tray.
- No, keep this instrument in the tray.

Section 5 – Instruments used in 10-20% of cases

For each instrument listed below, please indicate whether you agree with its removal from the tray:

24 Langenbeck retractor (60x20mm)

25 Allis tissue forceps, atraumatic (200mm)

26 Baby crile forceps

Original tray contains 6 pieces, suggestion: reduce to 4 pieces

- o Yes, remove 2 of these instruments from the tray.
- o No, keep all 6 pieces in the tray.
- o No, remove only 1 instead of 2 pieces.

27 Debakey dissecting forceps (180mm)

28 Halsted mosquito forceps

Original tray contains 6 pieces, suggestion: reduce to 4 pieces

- o Yes, remove 2 of these instruments from the tray.
- o No, keep all 6 pieces in the tray.
- o No, remove only 1 instead of 2 pieces.

29 Nissen artery curved forceps (185mm)

Original tray contains 4 pieces, suggestion: reduce to 1 piece

- o Yes, remove 3 of these instruments from the tray.
- o No, keep all 4 pieces in the tray.
- o No, remove only 1 instead of 3 pieces.
- o No, remove only 2 instead of 3 pieces.

30 Adson blunt retractor (200mm)

31 Metzenbaum scissors (145mm)

32 Scissors straight, blunt (145mm)

33 Adson surgical forceps with plateau (120mm) (2 pieces)

34 Bowl polypropylene 250cc

Original tray contains 3 pieces, suggestion: reduce to 2 pieces

- o Yes, remove 2 of these instruments from the tray.
- o No, keep all 3 pieces in the tray.
- o No, remove only 1 instead of 2 pieces.

35 Bowl polypropylene 60cc

Answer options for single instruments listed above:

- o Yes, remove this instrument from the tray.
- o No, keep this instrument in the tray.
