# Supplementary material for: Surgical tray optimization: a prospective and survey-based evaluation of environmental and economic outcomes
Source: Surg Endosc. 2026 Jan 23;40(4):3080–9. doi: 10.1007/s00464-025-12499-2 (PMC13053359; doi:10.1007/s00464-025-12499-2)
Supplement: Supplementary file 4 — Supplementary file4 (PDF 122 KB)—Appendix D: Inventory of the life cycle assessment for using the current instrument set to conduct one surgery [file 464_2025_12499_MOESM4_ESM.pdf]

Appendix D: Inventory of the life cycle assessment for using the current instrument set to conduct one surgery

For the alternative scenarios, the quantities in the manufacturing and end-of-life section will reduce by approximately 43%, 54%, 18%, 31% and 22% for the <10% unused, <20% used, total survey reduction, surgeon survey reduction and scrub nurses survey reduction respectively.

Manufacturing				
Input	*Quantity*	*Unit*	*Dataset*	*Source*
Stainless steel	8.61876	Grams	Self-modeled	[1, 2]
Metal working	8.61876	Grams	Metal working, average for chromium steel product manufacturing {RER}| cutoff, S	-
Transport	0.004921312	Tkm	transport, freight, lorry >32 metric ton, euro6 {RER} | market for transport, freight, lorry >32 metric ton, EURO6 | cutoff, S	Google Maps
Use phase				
Input	*Quantity*	*Unit*	*Dataset*	*Source*
Wind energy	172.7032424	Wh	electricity production, wind, 1-3MW turbine, onshore {DK} | cutoff, S	Technical service department, machine data sheets, hospital power plant, sterilization department
Gas energy	61.88288921	Wh	electricity production, natural gas, conventional power plant {NL} | cutoff, S	Technical service department, machine data sheets, hospital power plant, sterilization department
Pressurized air	0.013100444	M^3	market for compressed air, 1000 kPa gauge {RER} | cutoff, S	Technical service department, machine data sheets, sterilization department
Steam	2.540601865	Kg	market for steam, in chemical industry {RER} | cutoff, S	Technical service department, machine data sheets, sterilization department
Water (RO)	2.713321019	L	market for water, deionised {Europe without Switzerland} | cutoff, S	Technical service department, machine data sheets, sterilization department

Water (softened)	25.51494041	L	market for water, completely softened {RER} | cutoff, S	Technical service department, machine data sheets, sterilization department
Soap 1	0.4175	Ml	Self-modeled	Technical service department, sterilization department [3]
Soap 2	17.535	Ml	Self-modeled	Technical service department, sterilization department [4]
Soap 3	1.002	Ml	Self-modeled	Technical service department, sterilization department [5]
Bluewrap	102	G	market for textile, nonwoven polypropylene {GLO} | cutoff, S	Sterilization department
Wastewater	28.24721593	L	market for wastewater, average {Europe without Switzerland} | cutoff, S	-
End-of-life				
Input	*Quantity*	*Unit*	*Dataset*	*Source*
Medical waste bin (50L)	0.000430938	Piece	Self-modeled	National Institute for Public Health and the Environment
Medical waste incineration	8.619190938	Grams	Self-modeled	[6]
Plastic garbage bag	0.059466	Grams	packaging film, low density polyethylene {GLO} | market for | cutoff, S	-
Regular waste incineration	102.059466	Grams	process-specific burdens, municipal waste incineration {Europe without Switzerland} | market for process-specific burdens municipal waste incineration | cutoff, S	-
Transport	0.019454957	Tkm	transport, freight, lorry >32 metric ton, euro6 {RER} | market for transport, freight, lorry >32 metric ton, EURO6 | cutoff, S	Waste disposal companies, Google Maps

References

(1) What is surgical steel? The role of stainless in healthcare. **2022**.

(2) Stolarski, T.; Nakasone, Y.; Yoshimoto, S. Engineering Analysis with ANSYS Software. In *Elsevier eBooks*, Elsevier, 2006.

(3) *neodisher Dekonta Med*; 2024. https://www.drweigert.com/target/SDB/neodisher-Dekonta-Med_SDB_NLnl_PN4212_2024-03-11.PDF.

(4) *neodisher MediClean*; 2023. https://www.drweigert.com/target/SDB/neodisher-MediClean_SDB_NLnl_PN4043_2022-08-25.PDF.

(5) *neodisher MediKlar*; 2023. https://www.drweigert.com/target/SDB/neodisher-MediKlar_SDB_NLnl_PN4045_2022-03-18.PDF.

(6) Zhao, W.; van der Voet, E.; Huppes, G.; Zhang, Y. Comparative life cycle assessments of incineration and non-incineration treatments for medical waste. *The International Journal of Life Cycle Assessment* **2009**, *14* (2), 114-121. DOI: 10.1007/s11367-008-0049-1.
