# Supplementary material for: Surgical tray optimization: a prospective and survey-based evaluation of environmental and economic outcomes
Source: Surg Endosc. 2026 Jan 23;40(4):3080–9. doi: 10.1007/s00464-025-12499-2 (PMC13053359; doi:10.1007/s00464-025-12499-2)
Supplement: Supplementary file 5 — Supplementary file5 (PDF 74 KB)—Appendix E: Overview of assumptions for life cycle assessment [file 464_2025_12499_MOESM5_ESM.pdf]

Appendix E: Overview of assumptions for life cycle assessment

- The lifetime of the instruments is 500 usages (source: previous studies, sterilization department)
- Throughout their lifetime they are rarely repaired (hence its exclusion in the scope, see figure 2) (source: sterilization department)
- The instruments are manufactured in Tuttlingen (source: previous studies, [1])
- The thermodesinfector fits 12 surgical instrument trays (source: sterilization department)
- The sterilizer fits 16 surgical instrument trays (source: sterilization department)

References

(1) Smiskelly. *Tuttlingen, the world's surgical instrument capital*. 2017. https://www.usms.biz/tuttlingen-surgical-instrument-capital/ (accessed 2025 May).
