# Supplementary material for: Surgical tray optimization: a prospective and survey-based evaluation of environmental and economic outcomes
Source: Surg Endosc. 2026 Jan 23;40(4):3080–9. doi: 10.1007/s00464-025-12499-2 (PMC13053359; doi:10.1007/s00464-025-12499-2)
Supplement: Supplementary file 6 — Supplementary file6 (PDF 76 KB)—Appendix F: Types and frequencies of observed procedures [file 464_2025_12499_MOESM6_ESM.pdf]

Appendix F: Types and frequencies of observed procedures

	Number of procedures (N=162) (%)
Breast surgery	*77 (47.5%)*
Unilateral lumpectomy or mastectomy (including sentinel node)	48 (29.6%)
Unilateral lumpectomy or mastectomy (without sentinel node)	20 (12.3%)
Bilateral lumpectomy or mastectomy (including sentinel node)	7 (4.3%)
Bilateral lumpectomy or mastectomy (without sentinel node)	2 (1.2%)
Minor surgery	*19 (11.7%)*
Diagnostic biopsy	3 (1.9%)
Removal of lymphatic node	8 (4.9%)
Removal of melanoma, lipoma, or atheroma	8 (4.9%)
Endocrine surgery	*16 (9.9%)*
Hemi(para)thyroidectomy	12 (7.4%)
Total thyroidectomy	4 (2.5%)
Hernia surgery	*15 (9.3%)*
Open repair unilateral inguinal hernia (Lichtenstein)	8 (4.9%)
Open repair incisional or umbilical hernia	3 (1.9%)
Repair incisional or umbilical hernia with mesh	4 (2.5%)
Robotic surgery	*10 (6.2%)*
Robot assisted hemithyroidectomy	1 (0.6%)
Robot TME	1 (0.6%)
Robot-assisted colectomy	1 (0.6%)
Robotic (recto)sigmoid resection	6 (3.7%)
Robotic hemicolectomy	1 (0.6%)
Other	*25 (15.4%)*
Implantation or removal of sacral neuromodulator	19 (11.7%)
VAC (Vacuum-Assisted Closure) switch	1 (0.6%)
Sarcoma resection	3 (1.9%)
Incision and drainage of abscess	1 (0.6%)
Post operative bleeding control	1 (0.6%)
