# Supplementary material for: Surgical tray optimization: a prospective and survey-based evaluation of environmental and economic outcomes
Source: Surg Endosc. 2026 Jan 23;40(4):3080–9. doi: 10.1007/s00464-025-12499-2 (PMC13053359; doi:10.1007/s00464-025-12499-2)
Supplement: Supplementary file 7 — Supplementary file7 (PDF 84 KB)—Appendix G: Characteristics of staff surveyed for instrument review [file 464_2025_12499_MOESM7_ESM.pdf]

Appendix G: Characteristics of staff surveyed for instrument review

	**n (%)**
Gender	
Male	13 (27.7%)
Female	34 (72.3%)
Prefer not to say	0 (0%)
Prefer to describe myself as…	0 (0%)
Current function	
Resident	3 (6.4%)
Fellow	2 (4.3%)
Attending 0-5 years	3 (6.4%)
Attending 6-9 years	5 (10.6%)
Attending >10 years	7 (14.9%)
Scrub nurse in training	1 (2.1%)
Scrub nurse 0-5 years	7 (14.9%)
Scrub nurse 6-9 years	3 (6.4%)
Scrub nurse >10 years	16 (34.0%)
Working experience in this hospital	
0-5 years	14 (29.8%)
6-10 years	11 (23.4%)
>10 years	22 (46.8%)
Frequency of tray usage	
Daily	1 (2.1%)
Multiple times a week	14 (29.8%)
Weekly	16 (34.0%)
Multiple time a month	6 (12.8%)
Less than once a month	10 (21.3%)
Surgical discipline (medical doctors)	
General surgery	15 (75.0%)
Urology	2 (10.0%)
Plastic surgery	2 (10.0%)
Gynecology	1 (5.0%)
Specialized discipline (scrub nurses)	
Yello – general surgery	11 (40.7%)
Blue	5 (18.5%)
Green	7 (25.9%)
Red	4 (14.8%)
